# Echo-Endoscopy Combined with Virtual Reality: A Whole Perspective of Laparoscopic Common Bile Duct Exploration in Children

**DOI:** 10.3390/children10040760

**Published:** 2023-04-21

**Authors:** Francesca Destro, Raffaele Salerno, Valeria Calcaterra, Sandro Ardizzone, Milena Meroni, Margherita Roveri, Ugo Maria Pierucci, Alberta Zaja, Francesco Rizzetto, Alessandro Campari, Maurizio Vertemati, Paolo Milani, Gloria Pelizzo

**Affiliations:** 1 Department of Pediatric Surgery, “Vittore Buzzi” Children’s Hospital, 20154 Milan, Italy; francesca.destro@asst-fbf-sacco.it (F.D.);; 2 Gastrointestinal and Digestive Endoscopy Unit, ASST Fatebenefratelli Sacco, 20157 Milan, Italy; 3 Department of Pediatrics, “Vittore Buzzi” Children’s Hospital, 20154 Milan, Italy; 4 Department of Internal Medicine and Therapeutics, University of Pavia, 27100 Pavia, Italy; 5 CIMaINa (Interdisciplinary Centre for Nanostructured Materials and Interfaces), University of Milano, 20133 Milan, Italy; 6 Postgraduate School of Diagnostic and Interventional Radiology, University of Milan, via Festa del Perdono 7, 20122 Milan, Italy; 7 Department of Pediatric Radiology, “Vittore Buzzi” Children’s Hospital, 20154 Milan, Italy; 8 Department of Biomedical and Clinical Science, University of Milano, 20157 Milan, Italy

**Keywords:** ERCP, endoscopic US, laparoscopy (LCBDE), virtual reality, children

## Abstract

*Introduction:* Endoscopic procedures are performed more frequently in children due to technological advances that can be safely performed in an adequate setting with a support of a multidisciplinary team. Pediatric indications for ERCP (endoscopic retrograde cholangiopancreatography) and EUS (endoscopic ultrasound) occur mainly due to congenital malformations. In a pediatric case series, we report the application of EUS combined with duodenoscopy, eventually associated with ERCP and minimally invasive surgery, highlighting the importance of defining a tailored dedicated management pathway for each patient. *Patients and methods:* A series of 12 patients, managed at our Center in the last three years, were evaluated, and their management was discussed. *Results:* EUS was performed in eight patients and permitted the differential diagnosis of duplication cysts and the visualization of the biliary tree and pancreatic anatomy. ERCP was attempted in five patients: in one case, it permitted the preservation of pancreatic tissue, postponing surgery and in three patients, it was technically unfeasible. MIS (minimally invasive surgery) was performed in seven patients, two with laparoscopic common bile duct exploration (LCBDE). Precise anatomical definition and the possibility of surgical simulation and team sharing were evaluated under VR HMD (Virtual Reality Head Mounted Display) in four cases. *Conclusions:* Exploration of the common bile duct in children differs from that of the adult population and combines echo-endoscopy and ERCP. The integrated use of minimally invasive surgery in the pediatric area is necessary for the whole management perspective in complex malformations and small patients. The introduction in the clinical practice of a preoperative study with Virtual Reality allows a better survey of the malformation and a tailored treatment.

## 1. Introduction

Endoscopy is essential for diagnosing and treating gastrointestinal (GI) disorders in adult patients [1]. Since its introduction, it has been improved by remarkable technological advances that allowed its application also in the pediatric population [1,2].

Endoscopic retrograde cholangiopancreatography (ERCP) and endoscopic ultrasound (EUS) applications in pediatrics differ from those in adults [1,3,4]. Literature data suggest that ERCP and laparoscopic common bile duct exploration (LCBDE) can be safely and effectively performed in children with proper surgical skills, staff coordination, and procedural systematization [5]. Indications of ERCP and EUS procedures are infrequent in children. Unlike in adult patients, the most common indications in children are hepatobiliary and pancreatic diseases and gastrointestinal duplications, mainly due to anatomical anomalies [4]. The frequency of malignant diseases is smaller than in adult patients, and pediatric gastroenterologists have fewer opportunities of performing ERCP/EUS due to the small number of these pathologies in children [1]. On the other hand, pediatric gallbladder pathology leading to cholecystectomy has progressively increased in the last three decades. In children, ERCP and laparoscopic exploration of the common bile duct (LCBDE) can be performed safely and effectively. Still, recent literature suggests that LCBDE should be a method of choice in pediatric patients [6]. Skilling in pediatric patients has been supported by collaborating with pediatric gastroenterologists, pediatric surgeons, adult gastroenterologists, anesthesiologists, and radiology staff.

Concerns regarding the instrumentation size and the operator’s expertise arise in advanced endoscopic procedures [7,8,9]. Moreover, awareness of the long-term effects of radiation exposure is mandatory; lastly, adult gastro-duodenoscopes have elevated outer diameters that may damage surrounding structures and compress the airways in small infants [7]. For this reason, a limited utility of scopes and related devices is reported in small children and infants [8,9,10,11].

In the present case series, we report our recent experience using echo endoscopy and duodenoscopy to manage pediatric disorders, children, and small patients with hepato-biliary, pancreatic malformations, and congenital duplication cysts. Our primary goal was to confirm the applicability of those techniques in children. We highlight the need to adapt the instrumentation to fit the patients’ size to determine the best indications to perform duodenoscopy and echo-endoscopy in young patients. Moreover, as secondary outcomes, we would like to emphasize the importance of combining different innovative approaches, specifically minimally invasive surgery (MIS) techniques (laparoscopy, thoracoscopy) and virtual reality imaging modalities, to define the best management pathway for each patient.

## 2. Materials and Methods

### 2.1. Patients

A series of twelve patients, managed at our Department of Pediatric Surgery (Buzzi Children’s Hospital), were evaluated. We included pediatric patients with congenital duplication cysts of the foregut and pancreato-biliary abnormalities managed in the last three years. Patients with midgut and hindgut duplications and patients treated before the study time were excluded. Demographic, clinical, and radiological data, endoscopic and surgical details, and outcomes were recorded. Management details were discussed among a dedicated pediatric multidisciplinary team based on medical history and imaging (X-ray, US and MRI)/endoscopic results. In recent cases, the three-dimensional (3D) reconstruction simulation was performed to improve preoperative planning.

The study was retrospectively performed according to the Declaration of Helsinki. Patients/legal guardians were asked for consent for future participation in retrospective studies when acquiring consent for surgical procedures. During the follow-up, once the terms of the study were established, confirmation of consent was requested again, and the study was illustrated.

### 2.2. Methods

#### 2.2.1. Endoscopic Instrumentation

ERCP with a standard adult duodenoscope (Pentax Medical^®^, Italia S.r.l., ED34-i10T outer diameter of 11.6 mm, Cornaredo (MI) Italy) was used in patients weighing >10 kg. The instrument has an inner channel diameter of 4.2 mm. The instrument is an HD+ duodenoscope, ergonomically designed.

Endoscopic ultrasound (EUS) combines the endoscopic procedure with a sonographic evaluation of the intestinal wall and surrounding structures. We used the EG-3870UTK Linear-Array Ultrasound Gastroscope (Pentax Medical^®^, Italia S.r.l. Cornaredo (MI) Italy) with an insertion tube diameter of 12.8 mm for patients > 15 kg. This echoendoscope has an ultrasound transducer integrated into the tip of the scope and a convex scan system allowing interventional maneuvers (e.g., fine needle aspiration or stent placement) through a working channel, providing a 120° view. The relatively rigid tip contraindicates its use in tiny patients due to the possible risk of injuries. It is possible to insert mini probes (12–30 Mhz frequency range) through small working channels (2–2.8 mm) of standard endoscopes and use them in small children. Miniprobes allow good examination of superficial structures (mucosa and vasculature), but their performance in examining deeper structures is low. EBUS (Ultrasound Video Bronchoscope) devices (diameter 6.3–7.4 mm with 2–2.2 mm working channel) were used in children <15 kg. This is an excellent example of how endoscopic instrumentation is adapted to the child. However, some limitations remain, including reduced mobility and possible suction ineffectiveness.

#### 2.2.2. Three-Dimensional (3D) Models

MRI images were exported into DICOM files and loaded into 3D Slicer v.4.11 (https://www.slicer.org, last update 22 November 2022) [12], a free, open-source software that allows image segmentation, i.e., labeling anatomical structures in medical images to separate them from the background and each other. The radiological images were elaborated with semi-automatic segmentation based on established parameters using unique algorithms and manual refinements to correct possible errors. These models were zoomable and viewable from many viewpoints. Moreover, in contrast with traditional volume-rendering techniques, each model can be hidden or shown in transparency, allowing to focus on specific structures.

The final 3D scene was validated, compared with the radiological images, and evaluated closely with the medical team.

#### 2.2.3. Virtual Reality HMD Model

The obtained 3D models were loaded into an HMD (Head Mounted Display) Model through a Universal Serial Bus (USB) connection using an in-house developed plugin for 3D Slicer. Specifically, we used the Oculus Quest v.1 (META Inc., Menlo Park, CA, USA), an all-in-one HMD equipped with an OLED display with a 1440 × 1600 pixel per eye resolution and a refresh rate of 72 Hz.

A previously developed app [13] readapted for Oculus Quest provided the team with an immersive visualization of the 3D reconstruction inside a dedicated Virtual Reality Environment (VRE). All the experts involved in the patient’s care wore the HMD and could launch the app, choose a reconstruction and explore it (e.g., motion, rotation, zoom, and transparency mode). Selected images and videos were shared with the team on a computer equipped with a dedicated app to allow and stimulate a fruitful discussion and provide the best management approach [14,15].

#### 2.2.4. Preoperative Virtual Reality HMD Setup Evaluation

All the MRI images were reviewed using the patient-specific 3D models using the VRE. The images were oriented according to the patient positioning for MIS. The manipulation of images allowed the navigation towards critical anatomical structures (e.g., vessels, biliary tree), the definition of anatomical relationships, and surgical steps.

#### 2.2.5. Surgical Approach

The VR workstation was set up in the operative room (OR), and the 3D reconstructed images were shown on a monitor. An experienced pediatric surgeon with endoscopic expertise and an adult endoscopist performed the procedures. In selected patients, an interventional radiologist was involved, as well. Endoscopies and surgeries were carried out under general anesthesia. The patient lay supine, well secured to the operating table to allow position changes during the procedure. Enough space under the table was left to accommodate the X-ray machine for cholangiography. Trocar positioning included two operative 3 mm ports and one camera.

## 3. Results

### 3.1. Patients’ Data

Table 1 resumes the patients’ demographic, clinical data, and endoscopic and surgical details. In the last three years, we planned to perform ERCP or EUS in twelve patients (mean age 7.4 years, range: 20 days–16 years; mean weight 30.3 kg, range: 3.5–80 kg) with hepatobiliary pathologies (nine patients) and gastrointestinal duplication cyst (three patients). The endoscopic US was successfully performed in all three patients with congenital duplication cysts and one case of gallbladder duplication and multiple associated anomalies. ERCP was not completed in four instances for technical problems (size limitations, difficulty reaching the papilla, and low angle of view to obtain adequate histological sample). In one patient with a complex malformation (pt n. 12), a first attempt of failed choledochal stenting resulted in the external insertion of two biliary stents followed by steroid therapy and successful combined “rendez-vous” choledochal cannulation. LCBDE was performed as a primary procedure (two cases) or after ERCP failure (two cases). Preoperative evaluation with VR HMD was used in four patients before surgical management was decided.

### 3.2. Preoperative Planning and Operations

Before defining disease management, our multidisciplinary team discussed patients. The team included pediatric surgeons with endoscopic expertise, pediatric radiologists, adult endoscopists and pathologists, and oncologists. In recent cases (four patients), preoperative images have been elaborated to obtain VR 3D images. The 3D model was examined and used to manage complex issues. The accurate anatomical definition, specifically the biliary tree structure, stimulated the team discussion and favored decision-making. The adult endoscopist performed advanced endoscopic procedures, and a training program was implemented to achieve the complete autonomy of at least two pediatric surgeons shortly.

#### 3.2.1. Congenital Malformations (Duplication Cysts)

**Figure 1:**
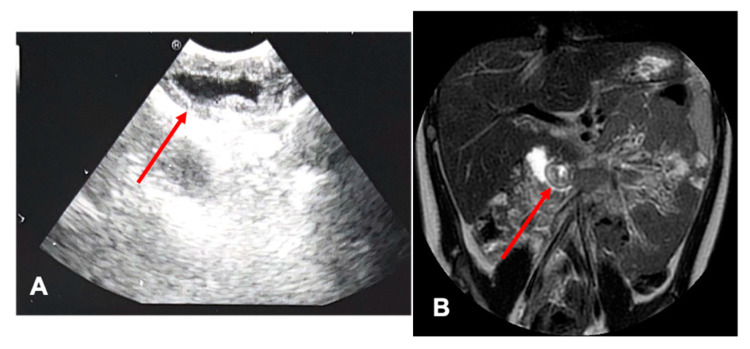
(**A**,**B**) Endoscopic US (**A**) and MRI (**B**) evaluation. Duodenal duplication in patient n. 7 (red arrows) with a cystic lumen surrounded by multiple layers, well evident on endoscopic US scan.

In patients 1, 2, 3, and 7, the endoscopic US was used before surgery to reduce the diagnostic possibilities (visualization of cysts surrounded by GI layers) and define the anatomy, Figure 1. Cyst removal by MIS (thoracoscopy and laparoscopy) was successfully performed in two patients at the age of 24 and 10 months, respectively. A 16-year-old girl is waiting for surgery; in her case, endoscopic US evaluation permitted to locate the pancreatic duct outlet precisely and to assess the feasibility of an endoscopic approach (cyst unroofing and mucosectomy).

#### 3.2.2. Biliary Tree Abnormalities

**Figure 2:**
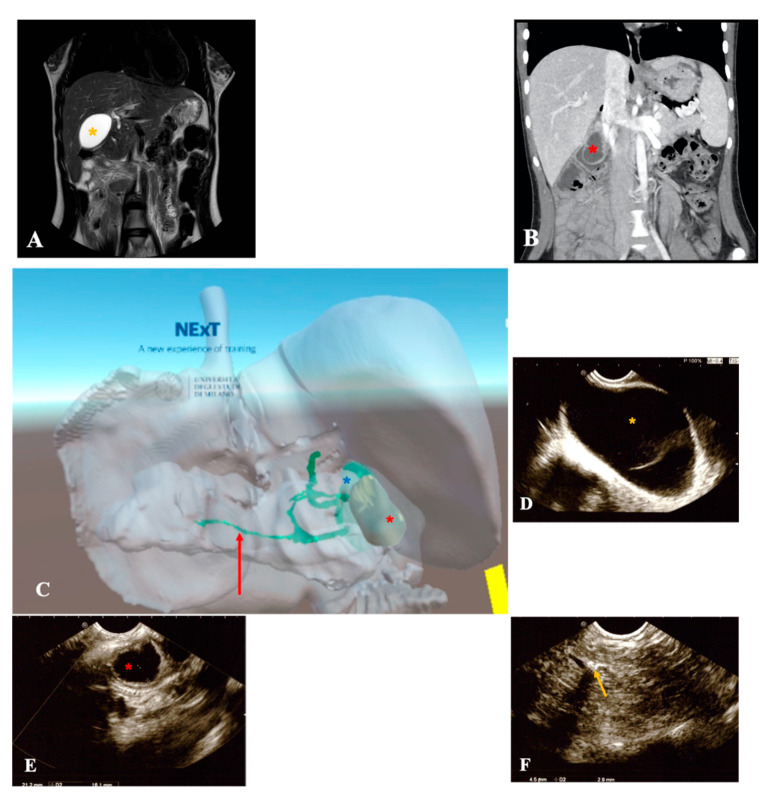
Patient n. 4 underwent surgery in the neonatal period for duodenal atresia. Years later, she developed symptomatic pancreato-biliary tree stones with cholelithiasis and choledocholithiasis. (**A**,**B**): Cholangio MRI showed a dilated gallbladder (yellow asterisk, (**A**)) with stones in the infundibulum and the choledochal channel near a cystic dilatation. (**D**–**F**): EUS confirmed the presence of a dilated gallbladder (yellow asterisk, (**D**)). We also identified a stone in the minor pancreatic duct (yellow arrow, (**F**)). (**C**): On the posterior view of the 3D reconstructions, the choledochal channel appears to end in the upper duodenal stump with a dilatation (blue asterisk in (**C**)). The pancreatic duct (red arrow in (**C**) ends in the lower duodenal stump. The upper duodenal stump also appears on endoscopic US as a blind-ending pouch (red asterisk in (**C**,**E**)).

**Figure 3:**
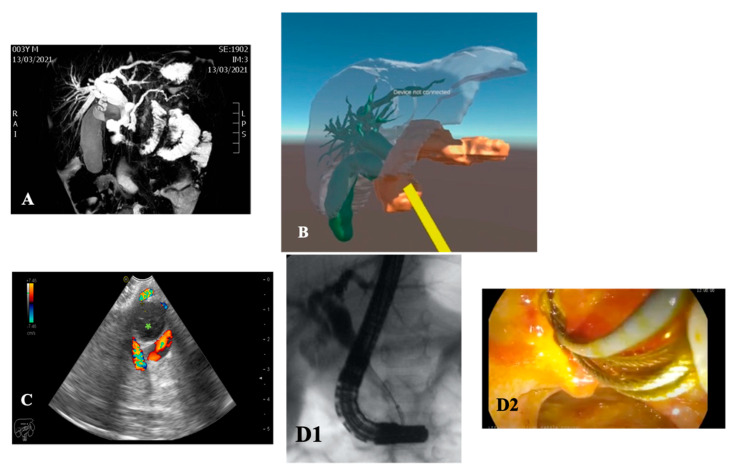
This patient with choledochal cyst (Todani I) and choledocholithiasis was admitted at the age of 4.3 years for cholestatic icterus and acute pancreatitis. (**A**,**B**): MRCP and 3D reconstructions showed a long common biliopancreatic channel (1.5–2 cm) with dilatation of the biliary tree and a pre-papillary stone. (**C**): Echoendoscopic evaluation (EBUS) confirmed the presence of a choledochal cyst (diameter 12.8 mm, green asterisk) and a 6 × 7 mm stone in the common biliopancreatic channel (17.9 mm). The main pancreatic duct was dilated (6.3 mm), and the accessory pancreatic duct appeared ecstatic (2.5 mm). (**D1**,**D2**): ERCP (endoscopic retrograde cholangiopancreatography) using the 11.6 mm duodenoscope with a lateral view; the choledocus appeared dilated in its middle portion as for type I Todani choledochal cyst with dilatation of the cranial biliary tree. The stone was removed after biliary sphincterotomy.

**Figure 4:**
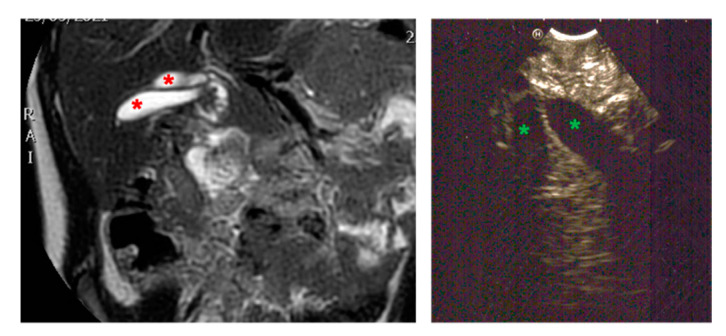
A duplicated gallbladder is seen on MRI scans (red asterisks) and intraoperative endoscopic US (green asterisks) in patient n. 7. The boy came to our attention after prenatal detection of the double gallbladder and annular pancreas. Cholangio MR showed two gallbladders that merged at the level of the proximal cystic duct and a cystic duodenal duplication between the second and third duodenum. The endoscopy showed mild duodenal stenosis below the first duodenum due to ab extrinsic compression by the annular pancreas. The US evaluation identified a normal choledochal channel (1.2 mm), an annular pancreas, and a cystic duodenal duplication (as shown in Figure 1) in the second duodenum. Biliopancreatic ducts ended up more distally. The gallbladder appeared duplicated with two cystic ducts that merged with the choledocus. No biliary dilatations were identified.

In three patients, lithiasis was associated with malformative conditions (n. 4 and 5, Figure 2 and Figure 3) and genetic predisposition (n. 6), for which preoperative evaluation (VR HMD and endoscopic US) was fundamental. In case 5, VR HMD permitted a faithful reproduction of the biliary tree by identifying an accessory branch unidentified with traditional imaging modalities. Patient 7 (Figure 4) identified multiple malformations: duplicated gallbladder, annular pancreas, and duodenal duplication. Preoperative endoscopic US study precisely defined the duplication features, excluding other differential diagnoses and assessing the caliber of the biliary tract.

#### 3.2.3. Proliferative Disorders

**Figure 5:**
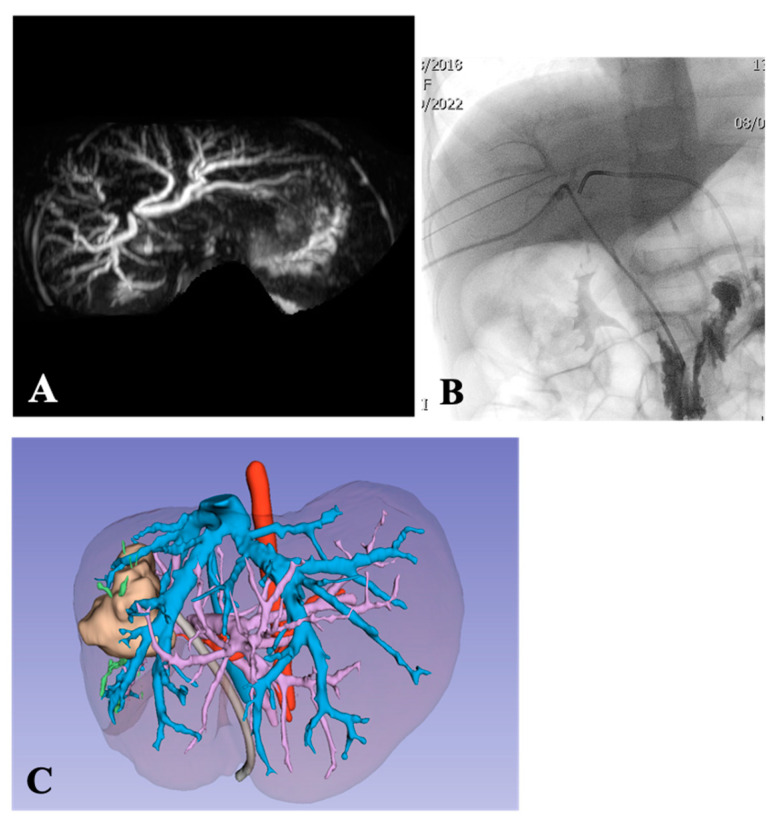
(**A**): Cholangio MRI of patient n. 12 showed a sizeable hepatic lesion of the right lobe extended from the subcapsular region to the hilum and determined biliary tree compression. The vascular branches form an intricate “caput medusae.” (**B**): Combined approach with percutaneous cholangiography and ERCP. The combined approach (Randez-vous) permitted to insert of a guidewire (0.035–400 mm), allowing a balloon dilatation (6 mm) and the stenting (8.5 F–12 cm). (**C**): VR reconstruction after removal of the left biliary stent. The intrahepatic right biliary tree could not be fully visualized due to complete obstruction of the anterior and posterior segments.

Two patients came to our attention with signs and symptoms of biliary tree obstruction. They were evaluated with combined 3D imaging and endoscopy for a complex picture of extensive hepatic involvement. Subsequently, samples were obtained by MIS in one of them, leading to the diagnosis of IMT (inflammatory myofibroblastic tumor). The management of the other patient was more complex due to severe edema and obstruction of multiple bile branches (Figure 5). Moreover, percutaneous biopsy specimens were inconclusive. A multi-step combined approach was adopted involving pediatric surgeons, adult endoscopists, interventional radiologists, and pediatric oncologists. Steroid therapy and choledochal stenting led to good clinical response.

### 3.3. Post-Operative Management

Post-operative management was set in such a way as to favor a rapid recovery of normal physiological functions, including oral feeding and early discharge. A nutritional program was implemented in collaboration with our pediatric dieticians and nutritionists. Serial ultrasounds were performed when required in the first postoperative days. Outpatient clinic appointments were scheduled based on personal needs.

## 4. Discussion

Adult gastrointestinal endoscopy (GIE) guidelines report the indications to perform EUS and ERCP, including tumors and gallstone disease [4]. These conditions are rare in children, and pediatric clinical reports are scarce. Pediatric GIE for hepato-biliary malformations has been progressively gaining importance in children [1,2,4]. The use of pediatric GIE has been favored by the development of miniaturized instruments and easy access to general anesthesia [7]. However, the literature data show that these procedures are performed only in a limited number of institutions, often with “borrowed instrumentation” provided by the manufacturer for each case on request [16]. On the other hand, dedicated pediatric scopes are frequently endowed with small-diameter working channels which may prevent the insertion of operative instruments [1,8,9].

Another theoretical boundary is represented by the fact that fewer procedures are performed in children than in adults. Adult endoscopists have more excellent “manual” experience in performing advanced GI endoscopic procedures, but we cannot refrain from guaranteeing specific and dedicated pediatric care. Children cannot be treated just as “smaller” adults; we should consider that they have different physiology and particular diseases [16,17,18]. In 2017, ESPGHAN (European Society for Pediatric Gastroenterology Hepatology and Nutrition), NASPGHAN (Nutrition and European Society of Gastrointestinal Endoscopy Guidelines), and ESGE (European Society of Gastrointestinal Endoscopy) reported pediatric gastrointestinal endoscopy guidelines, including indications to perform endoscopic procedures and the issue of endoscopist skills [4]. Considering duodenoscopy can be helpful in the case of hepatic, pancreatic, and duodenal pathologies; it can be associated with endoscopic ultrasonography, essential for ERCP (endoscopic retrograde cholangiopancreatography) [4,19,20,21]. However, in the era of “innovation-driven medicine,” excellence in medical care can only be achieved if we consider a multidisciplinary approach in which each one contributes with technological innovations specifically designed for the pediatric patient. In this perspective, endoscopic GI procedures should be integrated with preoperative 3D reconstructed images and MIS.

Indications for ERCP and EUS in children cover a broad spectrum of diseases, confirming the need for a multidisciplinary approach and the importance of a pediatric surgeon, especially in malformative conditions [4,21]. Our series of pediatric patients clearly show that EUS and duodenoscopy were performed primarily for congenital anomalies, especially in patients younger than five years of age. As expected, in these age ranges, the incidence of tumors and gallstone disease is lower than that in the adult population, and many congenital/anatomical disorders require surgery. Indications in adolescents are more similar to those of adults. However, we should also consider that there is a group of former “pediatric surgery patients” with gallstones or pancreatic duct anomalies related to the underlying congenital disease and to surgical outcomes (as we experienced in patient 4). ERCP and EUS may be effective in these patients, but they are associated with more significant technical difficulties and possible problems interpreting the results. The VR HMD may help overcome some of the challenges related to anatomical malformations given the possibility of obtaining exact anatomical images and guiding the surgeon during the operation, simulating the procedures, and preventing some complications.

In our experience, VR HMD was helpful in four complex cases (e.g., lithiasis with associated anatomical malformations and proliferative diseases) to visualize the biliary tree and define the intrahepatic involvement. It was an opportunity to share the operative approach and predict complications. The scene could be viewed simultaneously by all the team members, including surgeons, radiologists, endoscopists, and nurses, and represented an important team-building opportunity. In neonates and small infants, EUS was mainly indicated for diagnostic purposes, specifically gastrointestinal duplications [4,21]. EUS for gastrointestinal duplications permitted the differential diagnosis with other lesions, showing the typical features of a cyst surrounded by gastrointestinal wall layers. Together with the characterization of the lesion, EUS provided special relationships with surrounding structures allowing to tailor a specific therapeutic program in respect of the adjacent structures. In patients < 3 years of age, EBUS was applied successfully and represented a good example of a possibility of an instrument being adapted to fix the patient’s size. In older patients, EUS was an important diagnostic tool for pancreatic-biliary pathologies. It offered the possibility to define the anatomy better, especially the location of the papilla and its relationships with surrounding structures. It also helped assess the distal biliary tree. ERCP was performed as an additional approach in patient 2 to obtain a better anatomic view of the biliary system and to confirm the presence and location of stones before endoscopic treatment. According to our endoscopic societies (ESPGHAN, NASPGHAN, and ESGE), children have multiple indications to perform duodenoscopy, ERCP, and echoendoscopy [4,21]. Diagnostic ERCP is limited to selected cases with inconclusive imaging: negative MRCP (magnetic resonance cholangiopancreatography) in cholestasis or anomalous biliopancreatic junction for a prompt referral to surgery when required. Duodenoscopy and an operative approach can be performed if biliary and pancreatic pathologies require an ERCP for primarily therapeutic indications (e.g., biliary obstruction, pancreatic disease, ductal leaks, strictures). Echoendoscopy is considered for diagnostic purposes as an additional diagnostic modality after US and MRCP for pancreaticobiliary and GI lumen pathology. An associated therapeutic maneuver (EUS-guided drainage) can be performed in centers with specific expertise. In neonates and infants <1 year, ERCP has an almost exclusively diagnostic purpose. ERCP may help define the anatomy and identify patients with suspected biliary atresia that require an operation as soon as possible to delay liver transplantation and progression to biliary cirrhosis and end-stage liver disease [22,23]. The procedure may also exclude a congenital problem, avoiding unnecessary surgical procedures. Felux et al. performed 54 ERCPs in 31 children, including 6 with biliary atresia finding more detailed information on ductal integrity [24]. After demonstrating duct patency, these elements helped them plan and adapt the surgical approach and cancel the operation in 50% of cases [24]. Another ERCP indication in infants is pancreaticobiliary maljunction (PBM) evaluation in patients with suspected choledochal cysts and inconclusive radiological assessment (abdominal US, MRCP) [21,22,25]. The association with EUS may complete the evaluation [21]. There is also a possible role for preoperative 3D reconstructed images in these complex cases. In patients >1 year, operative ERCP is indicated for the complicated choledochal cyst to drain protein plugs and in complicated gallstone disease (choledocholithiasis, common bile duct dilatation, gallstone pancreatitis), to achieve biliary drainage or to remove the stone after endoscopic sphincterotomy [4,5,21,26]. However, recent papers suggest considering laparoscopic common bile duct exploration (LCBDE) to provide definitive treatment in a single procedure [5,27]. Congenital problems are still a frequent indication for ERCP in infants and children, while, similarly to adults, choledocholithiasis and malignant diseases are typical in adolescents [4,21]. Pediatric cholelithiasis is increasing in incidence, and cholesterol stones and biliary dyskinesia have emerged as main surgical indications [5]. According to Doud et al., the incidence of complicated pediatric disease due to cholesterol stones is 22.5% [28]. The dilatation of the common bile duct and common duct stones in these patients increases the risk and should indicate the need for ERCP [28]. ERCP reduces common bile duct explorations during surgery but is associated with a 5–10% complication risk [29,30,31]. In adults, total bilirubin > 4 mg/dL and common bile duct (CBD) dilatation > 6 mm are indicative of a recommendation for preoperative ERCP [32,33]. In children, these indicators seem less useful (ductal diameter does not correlate with the presence of calculi, and bilirubin > 4 mg/dl has low sensitivity for finding stones) [34,35]. Acute pancreatitis is considered by ASGE (American Society for Gastrointestinal Endoscopy) a risk factor for choledocholithiasis in adults [33]. However, recent studies in pediatric patients have not reported this correlation [35]. Specific pediatric algorithms are still missing. Recently, Capparelli et al. have proposed a risk score scheme to predict the presence of choledocholithiasis based on elevated total bilirubin, dilated CBD, and US detection of choledocholithiasis [36]. Given the non-standardization of CBD diameter in children, other authors propose to use conjugated bilirubin (and not total bilirubin as in the adult guidelines), gGT, AST, and ALT to improve the specificity of choledocholithiasis detection [28,37,38]. In adults with choledocholithiasis, EUS has a specific role in assessing the need for ERCP based on a risk stratification scheme, but its use in children is still less defined. EUS in pediatric microlithiasis can be considered in the case of symptomatic patients with negative transabdominal ultrasounds, and it allows ERCP to be performed during the same anesthesia [4,21]. Evidence suggests that retained stones are intraoperatively identified in the CBD after ERCP in up to 13% of patients [39]. LCBDE is an alternative strategy with the advantage of providing definitive treatment with a single procedure, limiting anesthesia, and having the likelihood of overcoming functional distal obstructions (e.g., sphincter spasm and sludge) by glucagon administration, ductal flushing or dilation [5]. Recently, Pogorelić et al. confirmed that LCBDE is safe and feasible in pediatric patients, avoiding papillotomy or fluoroscopy [6]. Therefore, the trend should be directed toward less ERCP in favor of LCBDE, provided LCBDE is performed in centers with dedicated teams and instrumentation [5].

**Figure 6:**
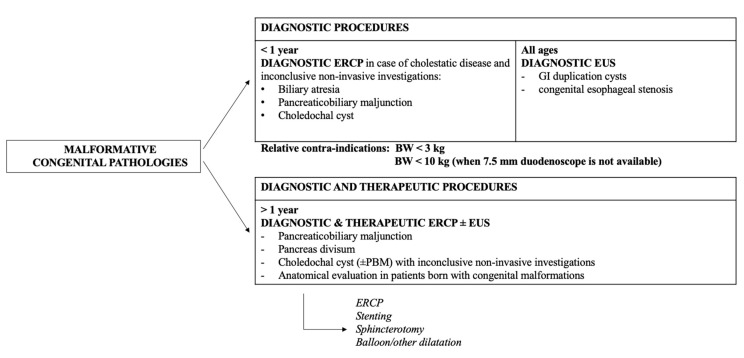

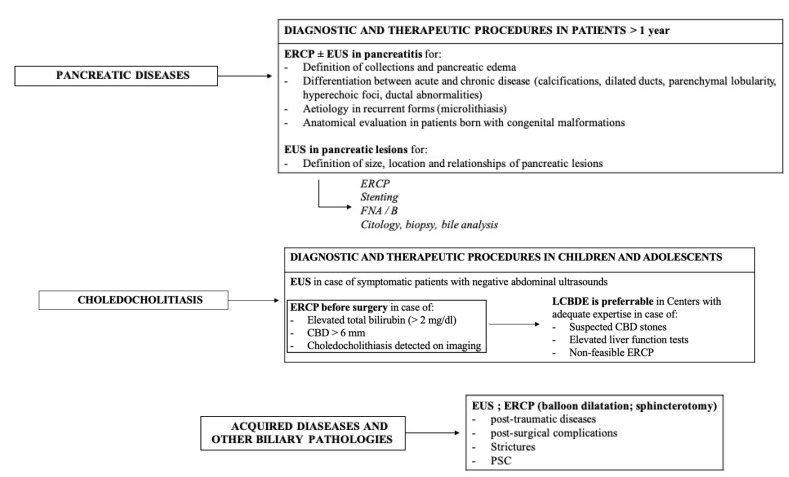
EUS and ERCP applications. PSC primary sclerosing cholangitis; PBM pancreaticobiliary maljunction; GI gastrointestinal; CBD common bile duct; LCBDE laparoscopic common bile duct exploration. They are modified based on ESPGHAN/ESGE/ASGE recommendations.

Figure 6 resumes diagnostic and therapeutic indications for EUS, ERCP, and LCBDE in cholelithiasis in our Pediatric Surgery Department at Children’s Hospital.

Data on pediatric duodenoscopy and EUS are scarce and primarily based on retrospective series lacking long-term follow-up [4]. ERCP has a reported success rate of 90.7% in children, comparable to that of adults (98%) [20]. Failure of the procedure seems related to young age and low body weight [40]. In neonates and small infants, ERCP is superior to MRCP in showing the main pancreatic duct and pancreaticobiliary junction features [41]. However, data on ERCP in these patients are mainly based on case reports and limited series. Only 3.3% of biliary atresia patients in Japan underwent ERCP in 29 years [23].

ERCP for cholelithiasis has the same efficacy and safety as LCBDE with no morbidity difference [5]. Moreover, LCBDE is associated with decreased length of stay and reduced number of procedures under general anesthesia. Post-endoscopic pancreatitis (PEP) occurs after ERCP in a percentage as high as 12% [42]. PEP prophylaxis with NSAIDs (diclofenac, indomethacin) should be considered in patients aged > 14 years, whereas there is no consensus for younger children. Other possible complications include bleeding (0.6%), infection (0.8%), and perforation [40]. Data on multidrug-resistant conditions by contaminated duodenoscopes have recently been reported (contamination rates are up to 23% of cases) [1,4,21]. New disposable instruments, such as detachable distal caps, disposable elevator caps, and single-use duodenoscope, have been proposed to overcome this problem but have the burden of high costs. Results on the safety of EUS duodenoscopies are reported for children > 15 kg (>3–4 years), although its application is evolving, also in smaller patients [21]. Piester et al. recently reported performing therapeutic EUS in children as small as 12 kg [3]. Their series of 72 patients (98 procedures) show a significant clinical impact of EUS, with 17.3% of cases that underwent endoscopic therapeutic interventions avoiding future surgeries.

In 2021, Lightdale et al. demonstrated that EUS is technically feasible and safe when performed by a pediatric gastroenterology-trained endosonographer [41]. The procedure helped them change the clinical management in 17.3% of their cases. Pediatric endoscopy is usually performed with techniques such as those used in adult patients. The literature data show that specific pediatric features may hamper the procedure’s success and that qualified operators are required to interpret diagnostic findings [1,4,42]. Duodenoscopy may be technically demanding in small children due to peculiar pediatric anatomy [20]. Firstly, neonates have esophagus and duodenum diameters measuring 5 and 10–15 mm, respectively, limiting the possibility of using large instruments that may compress the upper airways. The pylorus is challenging to visualize because the gastric antrum is acutely angulated. Using the prone position was proven helpful in minimizing respiratory discomfort.

Similarly, the angulation of the proximal duodenum hides the posteromedial wall. In these circumstances, maximum endoscopic tip deflection is required to proceed quickly through the gastrointestinal tract. Moreover, a very flexible tool is essential to adapt to tight spaces avoiding large loops that push over the intestinal walls.

Tagawa et al., have recently pointed out other unique issues of endoscopy in children, including specific emotional burdens and the need for anesthesia or deep sedation to perform the procedures [20]. The importance of a family- and patient-centered setting has also been emphasized in the clinical report of the Endoscopy and Procedures Committee, aiming at reducing anxiety and providing adequate sedation/analgesia [21,43].

The assisting nurses must be trained to assist both the endoscopist and the anesthesiologist but not simultaneously during the same procedure [21]. All these elements define the ideal location for patient management: a tertiary referral pediatric center with a consolidated collaboration network with adult endoscopists. We agree with Felux et al. that cooperation is essential before and after GIE and during the procedure [24]. The possibility of delivering high-quality endoscopic pediatric care is primarily based on the work of pediatric endoscopists with technical, cognitive, and integrative competencies producing a patient-tailored approach.

## 5. Conclusions

LCBDE may compensate for the difficulties encountered with endoscopy and in complex congenital malformations in children with previous surgery and may be considered superior to ERCP in the pediatric compared to the adult population. To define patient-tailored care, the pediatric surgeon may become more confident and skilled in answering the traps due to anatomic anomalies in small patients. The development of VR as a model of preoperative surgical simulations is suitable for a better understanding of the complexity of the anatomy. This preliminary experience underlies the importance of an early referral to a pediatric center with a dedicated team for common bile duct pathologies, including pediatric surgeons, pediatricians, adult gastroenterologists, radiologists, and trained nurses.

## Figures and Tables

**Table 1:** Data of patients, endoscopic and surgical details. EBUS endobronchial ultrasound; US ultrasounds; CT computed tomography; ERCP endoscopic retrograde cholangiopancreatography; MR magnetic resonance; DASE Dilation Assisted Stone Extraction; VR HMD Virtual Reality; LCBDE laparoscopic common bile duct exploration.

	Congenital Malformations (Duplication Cysts)			
	1	2		3
Patients age, weight	Female 24 months 12 kg	Female 10 months 10 kg		Female 180 months 59 kg
Symptoms	No	No		No
Diagnosis (prenatal evaluation yes/no)	Multiple gastric duplication Cyst (yes)	Esophageal duplication cyst (yes)		Duodenal duplication cyst
Radiological investigations	MR, US, EUS, X-ray	US, MR, CT-scan, barium swallow		US, MRI
Endoscopic instrumentation	EBUS	EBUS		EG-3870UTK Linear-Array Ultrasound Gastroscope
Management	MIS (laparoscopic resection of gastric duplication cysts)	MIS (thoracoscopic resection of esophageal duplication cyst)		Planned endoscopic removal by unroofing and mucosectomy
Endoscopic Advantages/Limitations	Reduction in the diagnostic possibilities by identification of cysts surrounded by gastrointestinal wall layers	Definition of the relationships with surrounding tissues		Anatomical definition of surrounding structures (in particular, the biliary tree and pancreatic duct)
	Biliary Tree Abnormalities Malformative/Congenital			
	4	5	6	7
Patients age, weight	Female 192 months 60 kg	Male 55 months 14 kg	Male 120 months 27 kg	Male 8 months 9 kg
Symptoms	Acute pancreatitis, pain in right hypochondrium	Icterus, pancreatitis	Chronic pancreatitis with choletithiasis, genetic-based	No
Diagnosis (prenatal evaluation yes/no)	Cholelithiasis and choledocholithiasis in duodenal atresia (duodeno-jejuno anastomosis at birth) and pancreas divisum (yes)	Choledochal cyst (Todani I) and choledocholitiasis (no)	Mutation of the gene PRSS1, Cholelitiasis, pancreatic duct duplication (no)	Gallbladder duplication Annular pancreas Duodenal duplication (yes)
Radiological investigations	US, MR, CT Scan, VR HMD	US, Cholangio MR	US, X-ray, Cholangio MR, CT, VR HMD	US, Cholangio MR, CT
Endoscopic instrumentation	EG-3870UTK Linear-Array Ultrasound Gastroscope	EBUS + Duodenoscope	Duodenoscope, EG-3870UTK Linear-Array Ultrasound Gastroscope	EBUS
Management	MIS (laparoscopic cholecisectomy) with LCBDE	1. ERCP + sphincterotomy + stone removal 2. Open surgery: choledochal cyst removal and Roux-en-Y bilio-digestive anastomosis	1. EUS + ERCP + stent placement + sphincterotomy + DASE; MIS (laparoscopic cholecystectomy) with LCBDE 2. ERCP and pancreatic stent replacement	MIS (laparoscopic cholecystectomies) with attempted LCBDE (failure for fibrosis)
Endoscopic Advantages/Limitations	Anatomical definition ERCP technically impossible for difficulties in reaching the papilla	Diagnostic and therapeutic procedures	Effective biliopancreatic drainage permitted postponed cholecystectomy and pancreatic preservation	Anatomical definition
	Biliary Tree Abnormalities Idiophatic Lithiasis			
	8	9		10
Patients age, weight	Male 192 months 80 kg	Female 144 months 58 kg		Male 20 days 3.5 kg
Symptoms	Acute pancreatitis	Abdominal pain		Cholestatic icterus
Diagnosis (prenatal evaluation yes/no)	Cholelitiasis (no)	Cholelitiasis (no)		Non-syndromic paucity of interlobular bile ducts (no)
Radiological investigations	US, cholangio-MR, CT Scan	US, cholangio-MRI		US
Endoscopic instrumentation	EG-3870UTK Linear-Array Ultrasound Gastroscope			N/A
Management	MIS (laparoscopic cholecystectomy) with LCBDE	MIS (laparoscopic cholecystectomy) with LCBDE		Anterograde cholangiography, hepatic biopsy
Endoscopic Advantages/Limitations				Size limitations; impossibility to perform ERCP

## Data Availability

Data supporting reported results are archived in the first author’s personal datasets.
